# LeftyA decreases Actin Polymerization and Stiffness in Human Endometrial Cancer Cells

**DOI:** 10.1038/srep29370

**Published:** 2016-07-11

**Authors:** Madhuri S. Salker, Nicolas Schierbaum, Nour Alowayed, Yogesh Singh, Andreas F. Mack, Christos Stournaras, Tilman E. Schäffer, Florian Lang

**Affiliations:** 1 Department of Cardiology, Vascular Medicine and Physiology, Eberhard Karls University of Tübingen, Germany; 2 Institute of Applied Physics, Eberhard Karls University of Tübingen, Germany; 3 Institute of Anatomy Eberhard Karls University of Tübingen, Germany; 4 Department of Biochemistry, University of Crete Medical School, Heraklion, Greece

## Abstract

LeftyA, a cytokine regulating stemness and embryonic differentiation, down-regulates cell proliferation and migration. Cell proliferation and motility require actin reorganization, which is under control of ras-related C3 botulinum toxin substrate 1 (Rac1) and p21 protein-activated kinase 1 (PAK1). The present study explored whether LeftyA modifies actin cytoskeleton, shape and stiffness of Ishikawa cells, a well differentiated endometrial carcinoma cell line. The effect of LeftyA on globular over filamentous actin ratio was determined utilizing Western blotting and flow cytometry. Rac1 and PAK1 transcript levels were measured by qRT-PCR as well as active Rac1 and PAK1 by immunoblotting. Cell stiffness (quantified by the elastic modulus), cell surface area and cell volume were studied by atomic force microscopy (AFM). As a result, 2 hours treatment with LeftyA (25 ng/ml) significantly decreased Rac1 and PAK1 transcript levels and activity, depolymerized actin, and decreased cell stiffness, surface area and volume. The effect of LeftyA on actin polymerization was mimicked by pharmacological inhibition of Rac1 and PAK1. In the presence of the Rac1 or PAK1 inhibitor LeftyA did not lead to significant further actin depolymerization. In conclusion, LeftyA leads to disruption of Rac1 and Pak1 activity with subsequent actin depolymerization, cell softening and cell shrinkage.

LeftyA, also known as endometrial bleeding-associated factor (EBAF), is a known regulator of stemness and embryonic differentiation^1^. It has previously been shown that LeftyA can reprogram cancer cells^2^ leading to inhibition of cell proliferation, stimulation of apoptosis and thereby, suppression of tumor growth^2^,^3^. Together, these lines of data indicate LeftyA is a strong suppressor of tumor cell activity^4^,^5^,^6^. LeftyA has a powerful negative effect on Na^+^/H^+^ exchanger 1(NHE1) activity^7^, which is expected to compromise survival of tumor cells^8^,^9^,^10^. Regulators of NHE1 activity include the small G protein ras-related C3 botulinum toxin substrate 1 (GTPase Rac1)^11^ which is a member of the Rho GTPases family. Rac1 is a key regulator of the actin cell cytoskeleton^12^ and promotes the formation of lamellipodia^13^, which is essential for cell motility^14^. The regulatory proteins of the actin cytoskeleton play a pivotal role for the motility of cancer cells and contribute to most steps during cancer progression^15^,^16^. The ability of cancer cells to invade the surrounding tissue, crossing the endothelial barrier to metastasize at a secondary site requires a highly dynamic reorganization of the actin cytoskeleton^17^. Rac1 and other Rho GTPases were found to be overexpressed in many types of cancer^18^,^19^,^20^. Down-regulation of Rac1 activity suppresses tumor growth and Rac1 was therefore identified as a potential therapeutic target for cancer cell treatment^21^,^22^. As the actin cytoskeleton provides the structural scaffold of a cell and mainly determines its mechanical properties^23^,^24^ alteration of actin polymerization is in turn anticipated to modify cell stiffness^25^,^26^.

Here we report that treatment of human endometrial carcinoma cells with LeftyA leads to dynamic change in mechanical cellular properties in tumor cells. We further provide evidence that in Ishikawa cells LeftyA decreases Rac1 activity, p21 protein-activated kinase 1 (PAK1) phosphorylation, actin polymerization, cell stiffness, area and volume.

## Results

### Impact of LeftyA on the stiffness and the shape of Ishikawa cells

We recently have shown that LeftyA can decrease expression and activity of the NHE1^7^. NHE1 in turn contributes to the stabilization and localization of actin. We hypothesized that NHE1 inhibition could alter the cytoskeleton necessary for maintaining cell structure. Filamentous actin (F-actin), a cytoskeleton protein known to have an important role in maintaining cellular and tissue structure^26^, is affected by changes in cytosolic pH (pH_i_)^27^.

**Figure 1:**
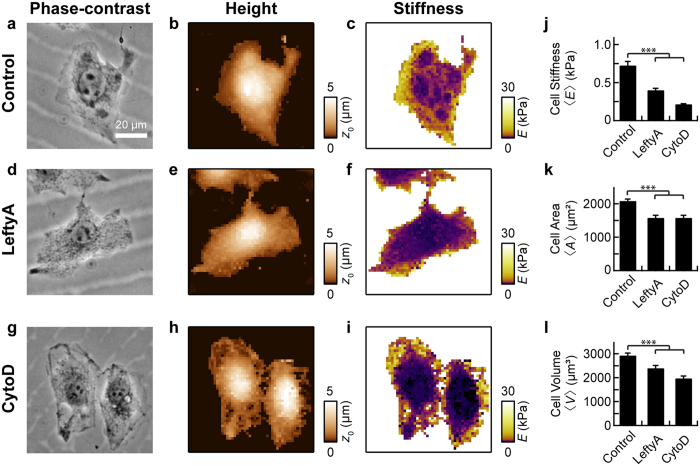
AFM analysis of stiffness and shape of Ishikawa cells with and without LeftyA treatment. Representative optical phase contrast image (**a**), AFM contact height image (**b**) and AFM stiffness image (**c**) of an untreated human endometrial cancer Ishikawa cell. Representative optical phase contrast image (**d**), AFM contact height image (**e**) and AFM stiffness image (**f**) of an Ishikawa cell treated for 2 hours with 25 ng/ml LeftyA. Representative optical phase contrast image (**g**), AFM contact height image (**h**) and AFM stiffness image (**i**) of an Ishikawa cell 15 min after addition of 10 μM cytochalasin D. Mean cell stiffness 〈*E*〉 (**j**), mean cell area 〈*A*〉 (**k**) and mean cell volume 〈*V*〉 (**l**) of untreated, LeftyA and cytochalasin D treated Ishikawa cells (〈*E*〉_Contr_ = 0.73 kPa, 〈*E*〉_LeftyA_ = 0.40 kPa, 〈*E*〉_CytoD_ = 0.21 kPa, 〈*A*〉_Contr_ = 2084 μm^2^, 〈*A*〉_LeftyA_ = 1579 μm^2^, 〈*A*〉_CytoD_ = 1580 μm^2^, 〈*V*〉_Contr_ = 2934 μm^3^, 〈*V*〉_Lefty*A*_ = 2403 μm^3^, 〈*V*〉_CytoD_ = 1974 μm^3^). Error bars represent SEM of geometric mean (**j**) and SEM of arithmetic mean (**k**,**l**). *P < 0.05; **P < 0.01; ***P < 0.001 using one-way ANOVA followed by two-tailed Tukey’s test.

To determine whether LeftyA impacts on cell shape and mechanical stiffness of human endometrial cancer Ishikawa cells, atomic force microscopy (AFM) was performed on live Ishikawa cells after a 2 hours treatment with LeftyA (25 ng/ml). The effect of LeftyA was compared to that of the cytoskeletal drug cytochalasin D, which induces rapid actin depolymerization. The cells exhibited large spatial variations of the local stiffness (Fig. 1c,f,i). The calculated single cell stiffness was averaged for a large number of cells to obtain a representative mean stiffness 〈*E*〉. LeftyA and cytochalasin D treated Ishikawa cells were significantly softer than the control cells (Fig. 1j; ***P = 1 × 10^−10^, ***P = 4 × 10^−9^). Histograms of the single cells stiffness values showed an approximately log-normal distribution, which was shifted to lower stiffness values for LeftyA and cytochalasin D treated Ishikawa cells, as compared to untreated Ishikawa cells (Supplementary Fig. S1). The width of the distribution was not affected by LeftyA but was significantly decreased by cytochalasin D (Supplementary Fig. S1; *P* = 0.35, ***P = 8 × 10^−6^). LeftyA- and cytochalasin D-induced softening was also observed for a single cell before and after treatment (Supplementary Figs S2 and S3).

LeftyA and cytochalasin D treated Ishikawa cells had a significantly lower mean cell area 〈*A*〉 (Fig. 1k; ***P = 7 × 10^−7^, ***P = 1 × 10^−4^) and a significantly lower mean cell volume 〈*V*〉 (Fig. 1l; ***P = 4 × 10^−6^, ***P = 0.001) as compared to the control cells. We also used optical phase-contrast microscopy over a large field of view to image multiple cells (Supplementary Fig. S4). The mean cell area significantly decreased after treatment with LeftyA (Supplementary Fig. S4; *P = 0.012). A decrease in cell area is consistent with our results using AFM. We note that the mean values are smaller compared to those obtained by AFM, owing to the known difficulties of cell contour tracking in optical phase-contrast images (e.g. Halo effect, similar brightness level of cell outlines and image background)^28^. AFM does not suffer from these limitations and gives more accurate results.

### Effect of LeftyA on actin polymerization in Ishikawa cells

**Figure 2:**
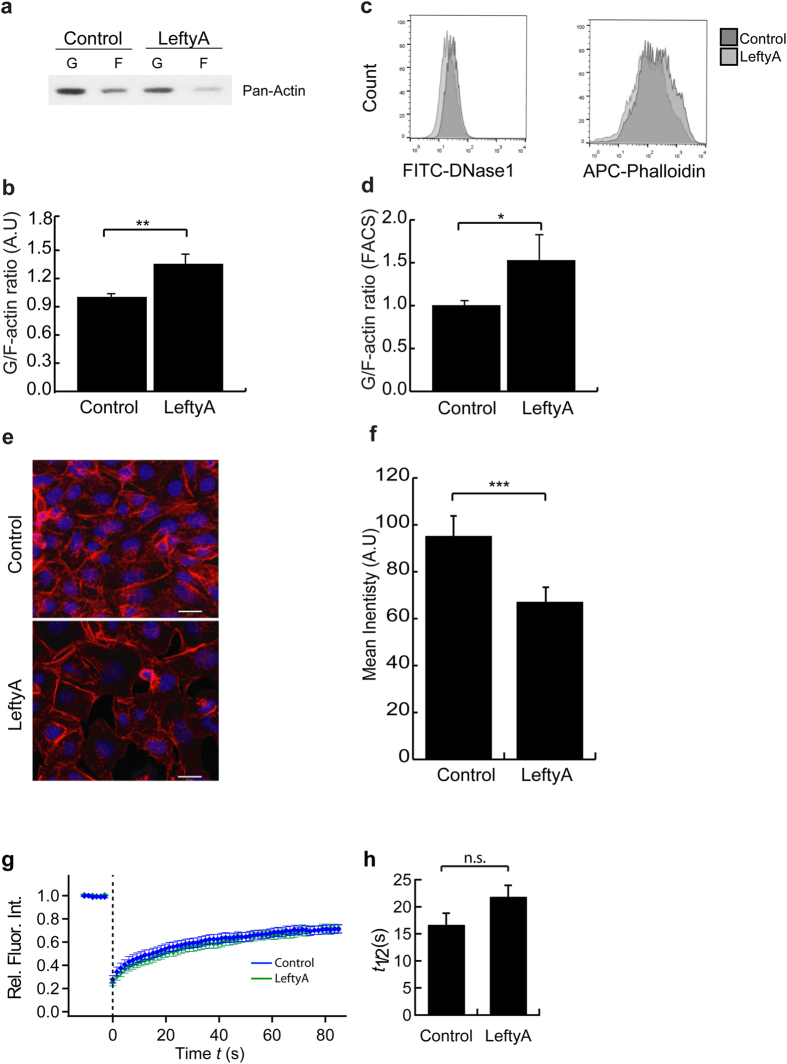
Effect of LeftyA on actin polymerization in Ishikawa cells. **(a)** Representative original Western blot of soluble G-actin over F-actin in human endometrial cancer Ishikawa cells after a 2 hour treatment without (−LeftyA) and with (+LeftyA) LeftyA (25 ng/ml). (**b)** Arithmetic means ± SEM (n = 6; arbitrary units) of soluble G-actin over F-actin ratio in Ishikawa cells after a 2 hour treatment without and with LeftyA (25 ng/ml). (**c)** Representative original histogram of DNase1 (G-actin; Left) and Phalloidin (F-actin; Right) binding in Ishikawa cells after a 2 hour treatment without (dark grey) and with (grey) LeftyA (25 ng/ml). (**d)** Arithmetic means ± SEM (n = 6; arbitrary units) of G-actin over F-actin ratio in Ishikawa cells after a 2 hours treatment without and with LeftyA (25 ng/ml). (**e)** Original confocal images of eflour660-phalloidin binding to F-actin (red) and SYTOX Green for nuclei (blue) in Ishikawa cells treated with or without LeftyA (white bar 20 μm) (**f**) arithmetic means  ±  SEM (*n*  =  6) of actin fluorescence in Ishikawa cells with and without LeftyA treatment. (**g**) Averaged FRAP curve (*n* = 21) of control cells or cells treated for 2h with LeftyA. Error bars denote SEM of arithmetic mean of normalized fluorescence intensity at each time point. (**h**) Mean half-time of recovery for LeftyA-treated (Green) and untreated Ishikawa cells (Control; Blue) obtained by FRAP. *P < 0.05; **P < 0.01; ***P < 0.001 using Student’s t-test.

We next explored whether the marked alterations of cell size, shape and stiffness following LeftyA treatment were paralleled by respective alterations of actin polymerization dynamics. Strikingly, as apparent from both Western blotting analysis (Fig. 2a,b; **P = 0.0087) and flow cytometry (Fig. 2c,d; *P = 0.048), a 2 hour treatment of Ishikawa cells with LeftyA was sufficient to significantly increase the amount of soluble G-actin over F-actin ratio, an observation reflecting depolymerization of the actin filaments. The effect of LeftyA was compared to that of the cytoskeletal perturbation drug cytochalasin D, which induces rapid actin depolymerization. Indeed in keeping with previous findings, treatment with cytochalasin D increased the amount of soluble G-actin over F-actin ratio reflecting depolymerization of the actin filaments (Supplementary Fig. S5). These findings were also replicated using HEK293 T-cells (Supplementary Fig. S5). These rapid effects were reversible upon washout (Supplementary Fig. S5) and may reflect an early and transient response of actin cytoskeleton dynamics in receiving and mediating extracellular signals as this was previously reported as well for cytokines, growth factors and steroid hormones^29^,^30^,^31^. Fluorescent images of F-actin organization and its concomitant changes under LeftyA show a profound reorganization of the actin cytoskeleton (Fig. 2e,f; ***P = 0.0007).

To investigate, whether LeftyA impacts the turnover rate of F-actin depolymerization/polymerization, we performed Fluorescence Recovery After Photobleaching (FRAP) on Ishikawa cells stably expressing Green Fluorescence Protein (GFP)-tagged F-actin. As shown in Fig. 2h, the half-time of recovery, which reflects the turnover reaction tended to be longer for LeftyA treated Ishikawa cells as compared to the control (*P* = 0.0869). However, the difference did not reach statistical significance.

### Effect of LeftyA on Rac1 transcript levels and activity in Ishikawa cells

**Figure 3:**
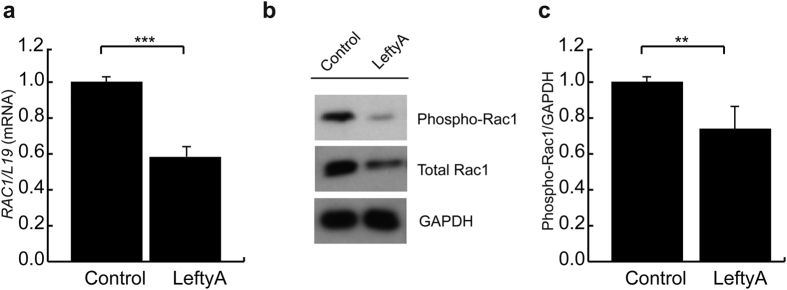
Effect of LeftyA on Rac1 transcript levels and activity in Ishikawa cells. (**a)** Arithmetic means ± SEM (n = 4) of Rac1 normalized to L19 transcript levels in human endometrial cancer Ishikawa cells following treatment with LeftyA (25 ng/ml) for 2 hours. Data are depicted as fold induction relative to transcript levels of untreated samples. (**b)** Representative original Western blots showing activated Rac1 and total Rac1 protein abundance in human endometrial cancer Ishikawa cells after 2 hours culture in the absence or presence of LeftyA (25 ng/ml). (**c)** Arithmetic means ± SEM (n = 4, arbitrary units) of phospho-Rac1 protein ratio normalized to GAPDH in Ishikawa cells after 2 hours culture in the absence or presence of LeftyA (25 ng/ml). *P < 0.05; **P < 0.01; ***P < 0.001 using Student’s t-test.

Small G protein or GTPase Rac1^11^ can regulate actin organization and polymerization^12^,^32^,^33^ thereby contributing to cell stiffness and cell volume^27^,^34^. In search for a cellular mechanism accounting for the reorganization of the actin filaments, the effect of LeftyA on Rac1 expression and activity was tested. As shown in Fig. 3a, LeftyA treatment (25 ng/ml for 2 h) was followed by a significant decline of Rac1 transcript levels normalized to L19 transcript levels (***P = 0.0035). Moreover, LeftyA treatment was followed by a significant decline of phospho-Rac1 (Fig. 3b,c; **P = 0.0094).

### Effect of LeftyA on actin polymerization in Ishikawa cells in absence or presence of Rac1 inhibitor

**Figure 4:**
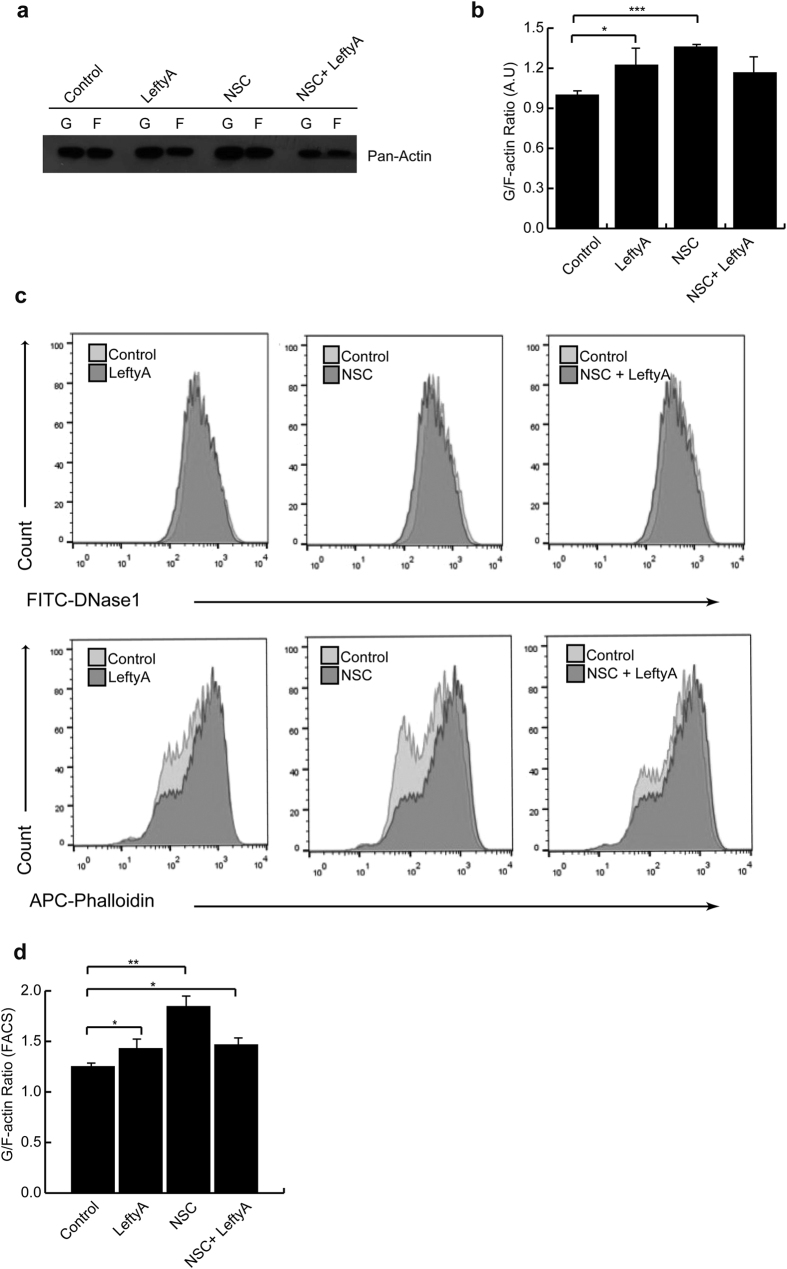
Effect of LeftyA on actin polymerization in Ishikawa cells in absence or presence of Rac1 inhibitor. **(a)** Representative original Western blot of soluble G-actin over F-actin in human endometrial cancer Ishikawa cells after a 2 hour treatment without and with LeftyA (25 ng/ml) in the absence and presence of the Rac1 inhibitor NSC23766 trihydrochloride (100 μM). (**b)** Arithmetic means ± SEM (n = 3; arbitrary units) of soluble G-actin over F-actin ratio in Ishikawa cells after a 2 hour treatment without and with LeftyA (25 ng/ml) in the absence and presence of the Rac1 inhibitor NSC23766 trihydrochloride (100 μM). (**c)** Representative original histogram of DNase1 (G-actin; Upper) and Phalloidin (F-actin; Lower) binding in Ishikawa cells after a 2 hour treatment without and with LeftyA (25 ng/ml) in the absence and presence of the Rac1 inhibitor NSC23766 trihydrochloride (100 μM). (**d)** Arithmetic means ± SEM (n = 5 arbitrary units) of G-actin over F-actin ratio in Ishikawa cells after a 2 hour treatment without and with LeftyA (25 ng/ml) in the absence and presence of the Rac1 inhibitor NSC23766 trihydrochloride (100 μM) *P < 0.05; **P < 0.01; ***P < 0.001 using Student’s t-test.

A further series of experiments tested whether the effect of LeftyA on Rac1 indeed contributed to the depolymerization following treatment with LeftyA (25 ng/ml) for 2 h. As illustrated in Fig. 4, application of the Rac1 inhibitor NSC23766 trihydrochloride (100 μM) for 2 hours was followed by a significant increase of soluble G-actin over F-actin in human endometrial cancer Ishikawa cells (***P = 0.0006), thus mimicking the effect of LeftyA treatment (*P *=* 0.037). In the presence of the Rac1 inhibitor the additional administration of LeftyA (25 ng/ml) did not lead to a significant further increase of the soluble G-actin over F-actin ratio in Ishikawa cells.

### Effect of Lefty A on PAK1 transcript levels and activity in Ishikawa cells

**Figure 5:**
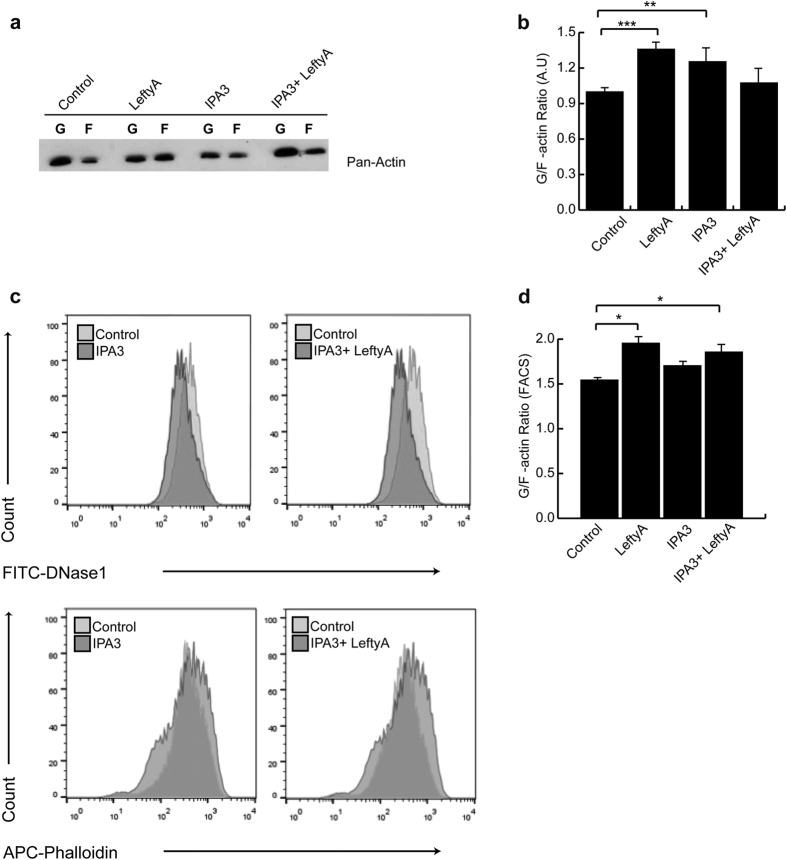
Effect of LeftyA on actin polymerization in Ishikawa cells in absence or presence of PAK1 inhibitor. **(a)** Representative original Western blot of soluble G-actin over F-actin in human endometrial cancer Ishikawa cells after a 2 hour treatment without and with LeftyA (25 ng/ml) in the absence and presence of the PAK1 inhibitor IPA-3 (50 μM). (**b)** Arithmetic means ± SEM (n = 6; arbitrary units) of soluble G-actin over F-actin ratio in Ishikawa cells after a 2 hour treatment without and with Lefty A (25 ng/ml) in the absence and presence of the PAK1 inhibitor IPA-3 (50 μM). (**c)** Representative original histogram of DNAse1 (G-actin; Upper) and Phalloidin (F-actin; Lower) binding in Ishikawa cells after a 2 hour treatment without and with LeftyA (25 ng/ml) in the absence and presence of the PAK1 inhibitor IPA-3 (50 μM). (**d)** Arithmetic means ± SEM (n = 5; arbitrary units) of G-actin over F-actin ratio in Ishikawa cells after a 2 hour treatment without and with LeftyA (25 ng/ml) in the absence and presence of the PAK1 inhibitor IPA-3 (50 μM). *P < 0.05; **P < 0.01; ***P < 0.001 using Student’s t-test.

Rac1 is known to trigger phosphorylation of PAK1, which in turn regulates the actin network^35^. As shown in Supplementary Fig. S6, application of LeftyA for 2 hours decreased PAK1 phosphorylation, pointing to a decrease of PAK1 activity in Ishikawa cells. Pharmacological inhibition of PAK1 with IPA3 (50 μM) again mimicked the destabilizing effect of inhibited Rac1 activity on the actin network (Fig. 5). Similar to inhibition of Rac1, inhibition of PAK1 was followed by a significant increase of soluble G-actin over F-actin in human endometrial cancer Ishikawa cells (Fig. 5b; **P = 0.008, Fig. 5d; *P = 0.034), an effect thus mimicking the effect of LeftyA treatment (Fig. 5b; ***P = 0.0007, Fig. 5d; *P = 0.045). In the presence of the PAK1 inhibitor the additional administration of LeftyA did not lead to a significant further increase of the soluble G-actin over F-actin ratio in Ishikawa cells.

## Discussion

**Figure 6:**
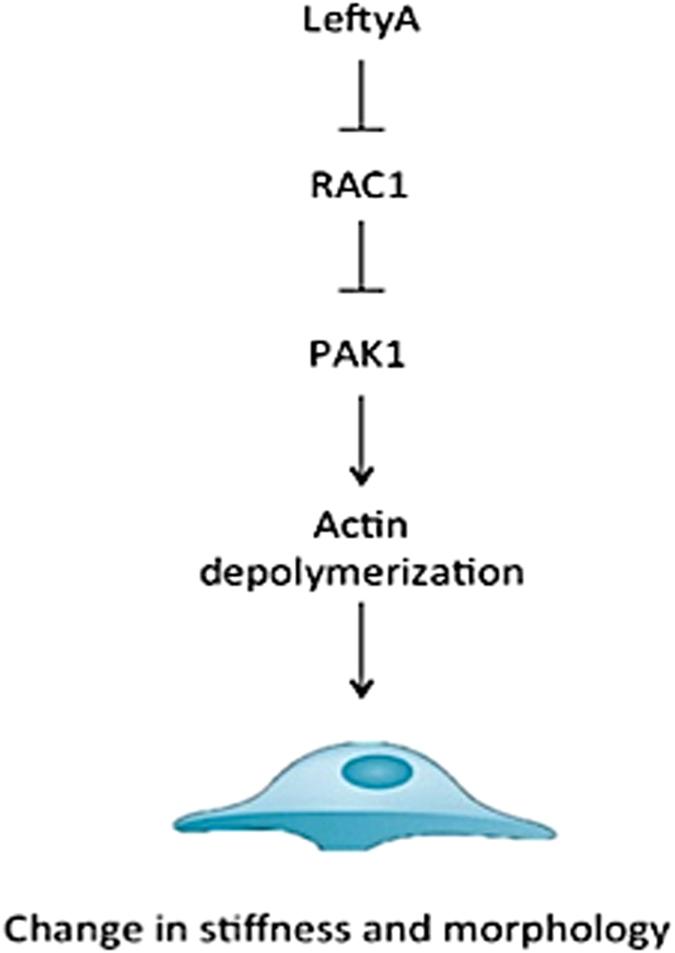
Schematic showing how stiffness of Ishikawa cells is affected by treatment with LeftyA via Rac1 and PAK1 pathways.

The present study describes a completely novel function of LeftyA in downregulating the expression and activity of the small G-protein Rac1 and of the kinase PAK1 with subsequent actin depolymerization as well as decrease of cell area, cell volume, and cell stiffness (Fig. 6).

As Rac1 is a key regulator of actin cytoskeleton organization^12^, down-regulation of Rac1 expression and activity following LeftyA treatment contributes to or even accounts for the observed actin reorganization, which in turn is expected to be followed by cell softening.

Rac1 activation is known to regulate actin polymerization interacting with the Arp2/3-mediated actin nucleation pathway rather than with the formin-mediated F-actin polymerization. On the other hand, it has been reported that the long formin-mediated F-actin accounts for the cortical elasticity of cells rather than the short Arp2/3-mediated F-actin^36^,^37^,^38^. In our study however, we observed a LeftyA-induced decrease of Rac1 (and PAK1) phosphorylation, indicating deactivation of these signaling effectors. In line with this, we observed reorganization of the actin filaments network towards depolymerization of F-actin filaments. Since modification of actin dynamics through depolymerization has been reported to account for filaments of lower mechanical stability^39^,^40^, our observations on cell softening in response to LeftyA treatment may directly be correlated to the observed LeftyA-induced actin depolymerization. Along those lines, depolymerization of the actin filaments by cytochalasin D was similarly followed by softening of the cells. In the presence of cytochalasin D, LeftyA failed to further soften the cells, an observation underscoring the role of the actin filaments in the softening effect of LeftyA. The present results are in line with previous observations in endothelial cells^26^,^41^,^42^. PAK1 phosphorylates actin, an effect resulting in actin depolymerization and redistribution of microfilaments^43^. In our results we show that treatment with LeftyA reduces both Rac1- and PAK1-activity. Inhibition of these two key molecules is further expected to decrease of lamellipodia formation^13^, which could explain the decrease of the cell area.

Regulators of actin polymerization^44^,^45^,^46^ and cell stiffness^47^ include the focal adhesion kinase (FAK)^32^, which activates several signaling molecules including Rac1^48^. Notably the signaling cascade triggered by FAK^49^,^50^ or Rac1^11^ further impacts on activity of NHE1. Thus, the presently observed inhibition of Rac1 may well contribute to the previously observed inhibition of the Na^+^/H^+^ exchanger by LeftyA^7^.

The observed decrease of cell volume may be secondary to inhibition of NHE, as stimulation of Na^+^/H^+^ exchange in parallel to Cl^−^/HCO_3_^−^ exchange increases cell volume^51^,^52^. The respective carriers accomplish entry of NaCl in exchange for H^+^ and HCO_3_^−^, which are replenished from CO_2_ and thus are not osmotically relevant^51^,^52^. Increase of cell volume is required at some stage for cell proliferation^51^,^52^. Alterations of cell volume require reorganization of actin filaments^53^,^54^, which in turn impact on cell stiffness.

In conclusion, the present study demonstrates that LeftyA down-regulates expression and function of the small G-protein Rac1 and of PAK1 with subsequent depolymerisation of the actin filaments. The reorganization of the actin filaments decreases cell stiffness and actin depolymerization and/or NHE inhibition decrease cell volume. Thus, this new role of LeftyA may provide new avenues to pursue the development of novel cancer therapeutics.

## Materials and Methods

### Cell Culture

Ishikawa cells, a well differentiated endometrial carcinoma cell line, or HEK293T cells were cultured in DMEM/F12 without phenol red media, containing 10% fetal bovine serum (FBS), 1% antibiotic/antimycotic solution and 0.25% L-Glutamine (Invitrogen, Karlsruhe, Germany). Cells were treated as described with recombinant human LeftyA (25 ng/ml; 746-LF-025/CF) (R&D Systems, Oxford, UK), Rac1 inhibitor (100 μM;NSC23766 trihydrochloride; Sigma Aldrich, München, Germany), PAK1 inhibitor IPA 3 (50 μM Tocris bioscience, Germany) or with the actin cytoskeleton-disrupting agent Cytochalasin D (10 μM, Sigma, Germany).

### Quantitative Real-time PCR

Total RNA was extracted from Ishikawa cultures using Trizol (Invitrogen) based on a phenol-chloroform extraction protocol. Equal amounts of total RNA (2 μg) were reverse transcribed by using the Superscript III First-Strand synthesis system for RT-PCR (Invitrogen) using an oligo dT primer and the resulting cDNA used as template in qRT-PCR analysis. The gene-specific primer pairs were designed using the Primerblast (NCBI) software. L19 was used to normalize for variances in input cDNA. Detection of gene expression was performed with KappaFast -SYBR Green (Peqlab, Erlangen, Germany) and quantitative RT-PCR was performed on a BioRad iCycler iQ Real-Time PCR Detection System (Bio-Rad Laboratories, München, Germany). The expression levels of the samples were expressed as arbitrary units. All measurements were performed in triplicate. Melting curve analysis and agarose gel electrophoresis confirmed amplification specificity.


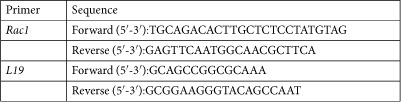


### Measurement of the G/F actin ratio by Triton X-100 fractionation

To quantify actin polymerization in Ishikawa cells, cells were incubated in 100 μl of Triton X-extraction buffer containing 0.3% Triton X-100, 5 mM Tris [pH 7.4), 2 mM EGTA, 300 mM sucrose, 2 μM phalloidin, 1 mM PMSF, 10 μg/ml leupeptin, 20 μg/ml aprotinin, 1 mM sodium orthovanadate, and 50 mM NaF for 5 min on ice. The supernatant containing the soluble proteins was removed by aspiration. The Triton X-insoluble pellet was scraped from the plate directly into 100 μl of RIPA buffer. Any remaining insoluble material was removed by centrifugation. Equal volumes of each fraction were boiled in Læmelli Buffer protein loading buffer at 95 °C for 15 min. Proteins were separated on 12% SDS-polyacrylamide gels and transferred to PVDF membranes. Nonspecific binding sites were blocked by overnight incubation with 5% nonfat dry milk in Tris-buffered saline with 1% Tween (TBS-T; 130 mmol/L NaCl, 20 mmol/L Tris, [pH 7.6] and 1% Tween). The membranes were incubated overnight at 4 °C with monoclonal rabbit anti-β-Actin (13E5)-HRP conjugated antibody (1:1000, Cell Signalling, Frankfurt, Germany). Antibody binding was detected with the Novex ECL Chemiluminescent Substrate Reagent Kit (Invitrogen) and bands were quantified using ImageJ software.

## Western Blotting

Whole cell protein extracts were prepared by lysing cells in RIPA buffer. Protein yield was quantified using the Bio-Rad DC protein assay kit (Bio-Rad, München, Germany). Equal amounts of protein were separated by 10% SDS-Polyacrylamide Gel Electrophoresis (SDS-PAGE) before wet-transfer onto PVDF membrane (Amersham Biosciences, UK). Nonspecific binding sites were blocked for 1 hour at room temperature with 5% nonfat dry milk in TBS-T. Membranes were probed overnight at 4 °C with an antibody against anti- RAC1, anti-phospho RAC1 and anti- GAPDH (Cell Signalling, Leiden, The Netherlands). All primary antibodies were used at 1:1000, washed 3 times with TBS-T, followed by incubation with HRP-conjugated anti-rabbit or anti-mouse secondary antibodies (P0448 and P0447, respectively; Dako, Ely, UK). Protein complexes were visualized with a chemiluminescent detection kit (Novex ECL Chemiluminescent Substrate Reagent Kit; Invitrogen) and bands were quantified using ImageJ software.

### G/F actin ratio by Flow cytometry

Ishikawa cells or HEK293T cells (≈1.0 × 10^5^ cells) were first fixed with 4% paraformaldehyde (PFA) and then permeabilised with 1x Permeabilization buffer (eBioscience, Frankfurt, Germany) and subsequently stained with 1.0 μl of fluorescent DNase1-Alexaflour-488 (50 mg/ml) for detection of G-actin and fluorescent Phalloidin-eFluor 660 (1000x) (eBioscience, Frankfurt, Germany) for detection of F-actin. The abundance of the respective labels was measured using green (FL-1) and red channel (FL-4) on a FACSCalibur (BD Biosciences, Heidelberg, Germany) and analysis was performed using Flowjo software (Flowjo LLC, Oregon, USA). G- and F-actin geometric mean values were determined from the respective fluorescence and the ratio of G/F calculated from the geometric mean values.

### AFM force mapping

AFM experiments on live Ishikawa cells were carried out with a commercial AFM setup (MFP3D Bio, Asylum Research, Santa Barbara, USA) 12 hours after the cells were seeded on fibronectin-coated (0.75 μg/cm^2^) culture dishes at a density of 0.5 × 10^4^ cells/cm^2^. 2 hours before AFM experiments, Ishikawa cells were treated with either 25 ng/ml LeftyA or PBS as vehicle control. Prior to the measurements, the cell culture medium was replaced with CO_2_-independent Leibovitz L-15 medium (Biochrom GmbH, Berlin, Germany), containing either 25 ng/ml LeftyA for the treated cells or PBS for the control cells. For the AFM experiments with cytochalasin D, Ishikawa cells were imaged 15 min after adding 10 μM (final concentration) cytochalasin D to the L-15 medium. The AFM experiments were performed at 37 °C.

Single Ishikawa cells were imaged with AFM in the force mapping mode^55^ (Supplementary Fig. S7). Maps of 50 × 50 force-indentation curves (maximum force 0.5 nN, tip-velocity 20 μm/s) on a 80 × 80 μm^2^ scan area were recorded using a single sphere-tip cantilever (Nanosensors SD-S-CONT-M, NanoWorld, Neuchâtel, Switzerland) with a nominal tip radius of *R* = 1 μm. The cantilever’s spring constant was determined by the thermal noise method^56^ as 0.33 N/m. To generate images of height and local stiffness (in terms of the elastic modulus *E*), the force-indentation curves were fitted with the spherical Hertz model^57^:


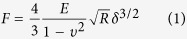


here, *F* is the measured force, *δ* the sample indentation and *ν* the Poisson ratio, which was assumed as 0.5 to model an incompressible sample. Representative force-indentation curves at different conditions are shown in Supplementary Fig. S7. The stiffness depends on the slope of the force-indentation curve. A steeper slope corresponds to a stiffer cell region. We averaged the local stiffness values within the cell area to obtain a more robust measure of single cell stiffness. To minimize the influence of the underlying substrate, only stiffness values for cell regions with a height above 1 μm were considered. The single cell area *A* was obtained by multiplying the number of pixels *N* within the cell outer contour with the calibrated pixel area *A*_px_, *A* = *N* · *A*_px_. The single cell volume *V* was obtained as the sum of the height values *h* at each pixel multiplied by the calibrated pixel area *A*_px_, 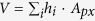. For 30 untreated control Ishikawa cells, for 30 LeftyA treated and for 13 cytochalasin D treated Ishikawa cells, the single cell stiffness values, areas and volumes were averaged to obtain representative mean values 〈*E*〉, 〈*A*〉, and 〈*V*〉, respectively. The single cell stiffness and the mean stiffness 〈*E*〉 were calculated as the geometric mean, because the elastic modulus of live cells follows a log-normal distribution^58^,^59^. The mean cell area 〈*A*〉 and the mean cell volume 〈*V*〉 were calculated as arithmetic means.

### Confocal microscopy

Ishikawa cells grown on chamber slides were fixed for 15 min with 4% paraformaldehyde, washed with PBS and permeabilized for 10 min in 0.1% Triton X-100/PBS. The slides were blocked with 5% goat serum in 0.1% Triton X-100/PBS for 1 hour at RT. Actin was stained with eflour660-phalloidin (1:200, Invitrogen) for 1 hour at RT and with SYTOX Green dye (1:5000, Invitrogen) for nuclei staining for 30 min in the dark. The slides were mounted with ProLong Gold antifade reagent (Invitrogen). Confocal microscopy was performed with a confocal laser-scanning microscope (LSM 5 Exciter, Carl Zeiss, Germany) with a C-Apochromat 63/1.3 NA DIC water immersion objective. The mean fluorescence from six related cells of each picture was quantified by ZEN software (Carl Zeiss, Germany).

### Fluorescence Recovery After Photobleaching (FRAP)

Ishikawa cells were grown on glass culture dishes and were transfected with CellLight Actin-GFP (F-actin), BacMam 2 (Invitrogen) for at least 16 h. FRAP experiments were performed on a Nikon C2 confocal microscope (Nikon, Japan) using a 100×/1.45 NA immersion oil objective. The system was equipped with a heating and an incubation system (ibidi, Germany) to maintain cells at 37 °C and 5% CO_2_. GFP fluorescence was excited at 488  nm. During the FRAP experiment a time-lapse sequence of images (256 × 256 pixels) of transfected Ishikawa cells were acquired. The pixel dwell time was set to 9.6 μs. Images were acquired in intervals of 1.5 s. Photobleaching a circular region of interest (ROI) with 3 μm in diameter was initiated after six images by setting the laser to its maximum power (corresponding to 15 mW at the end of the optical fiber) for approximately 1 s. 57 consecutive images were then collected in approximately 1.5 minutes during fluorescence recovery. The mean fluorescence intensities in the circular bleached ROI (diameter: 3 μm), in the circular unbleached control region (diameter: 3 μm) and in the circular background (bkGD) region (diameter: 5 μm) were measured (Supplementary Fig. S8).

FRAP curves were corrected for background fluorescence *F*_b_ and photofading and were normalized^60^


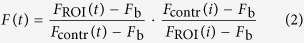


here, *F*_ROI_(*t*) and *F*_contr_(*t*) are the fluorescence intensities in the bleached ROI and in an unbleached region on the cell, respectively. *F*_ROI_(*t*) and *F*_contr_(*t*) are the initial pre-bleached fluorescence intensities in the bleached ROI and in the unbleached region, respectively. The time *t* corresponds to the time after the bleaching event occurred. Individual FRAP curves were fitted by a one-phase exponential equation (Supplementary Fig. S8):


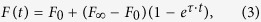


where *F*_0_ is the normalized fluorescence intensity immediately after bleaching in the ROI. The fluorescence intensity after recovery in the ROI, *F*_∞_, and the exponential decay parameter *τ* were used as free fit parameters. The half-time of recovery *t*_1/2_ was then calculated as 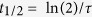. The arithmetic mean of the half-time of recovery for LeftyA treated and untreated Ishikawa cells was obtained by averaging *t*_1/2_ -values of 21 FRAP curves for each group.

### Statistics

Data are provided as means ± SEM, *n* represents the number of independent experiments. All data were tested for significance using one-way ANOVA followed by Student’s unpaired two-tailed *t*-test, Tukey’s test, or F-test. Only results with *P* < 0.05 were considered statistically significant.

## Additional Information

**How to cite this article**: Salker, M. S. *et al*. LeftyA decreases Actin Polymerization and Stiffness in Human Endometrial Cancer Cells. *Sci. Rep.* **6**, 29370; doi: 10.1038/srep29370 (2016).

## Supplementary Material

Supplementary Information
