# Supplementary material for: LeftyA decreases Actin Polymerization and Stiffness in Human Endometrial Cancer Cells
Source: Sci Rep. 2016 Jul 11;6:29370. doi: 10.1038/srep29370 (PMC4941646; doi:10.1038/srep29370)
Supplement: Supplementary Information [file srep29370-s1.pdf]

Supplementary Information

LeftyA decreases Actin Polymerization and Stiffness in Human Endometrial Cancer Cells

Madhuri S Salker[1]*, Nicolas Schierbaum[2]*, Nour Alowayed[1]*, Yogesh Singh[1], Andreas F Mack[3],Christos Stournaras[4#], Tilman E Schäffer[2#], Florian Lang[1#]

[1]Department of Physiology I, [2]Institute of Applied Physics, [3]Institute of Anatomy Eberhard Karls University of Tübingen, Germany, Eberhard Karls University of Tübingen, Germany and [4]Department of Biochemistry, University of Crete Medical School, Heraklion, Greece.

*Contributed equally and thus share first authorship.

[#]Contributed equally and thus share last authorship.

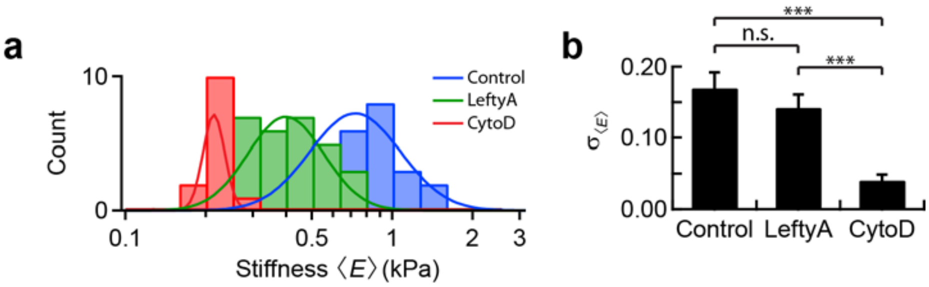

Figure Supplementary 1: Histograms of the mean stiffness values for single cells and the width of the stiffness distribution.

Distribution (a) and its width (defined as the geometric standard deviation) (b) of the stiffness of untreated, LeftyA treated and cytochalasin D treated Ishikawa cells (corresponding to the mean values shown in Fig. 1j). LeftyA treatment of Ishikawa cells shifts the stiffness distribution to lower values (a), but has no significant influence on the distribution width, compared to untreated cells (b). Addition of cytochalasin D not only shifts the stiffness distribution to lower values (a), but also significantly decreases the distribution width (b), compared to untreated and LeftyA treated Ishikawa cells. Error bars represents geometric standard error of the standard deviation. The solid traces in the histogram show the log-normal distributions. Data were tested for significance using F-test (*,$P < 0.05$; **, $P < 0.01$; ***, $P < 0.001$).

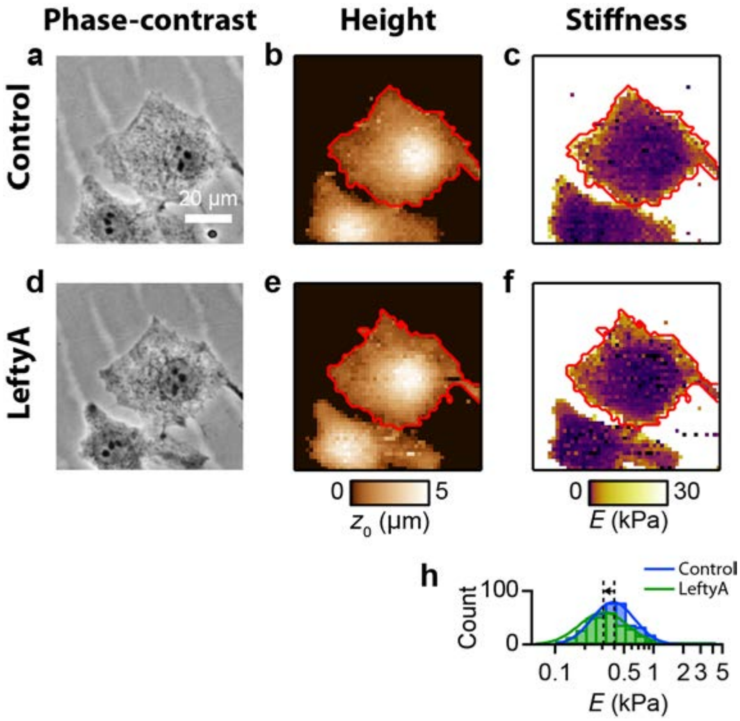

Figure Supplementary 2: AFM height and stiffness map of the same Ishikawa cell before and after 2h treatment with LeftyA.

Optical phase-contrast image, AFM contact height image and AFM stiffness image of Ishikawa cell before (a-c) and 2 hours after addition of LeftyA (d-f). The histogram of stiffness *E* (h) of the red outlined Ishikawa cell in (b,c) and in (e,f) shows a shift of the stiffness distribution after LeftyA treatment, corresponding to a decrease in mean stiffness by 20%. The solid traces in the histogram show the log-normal distributions.

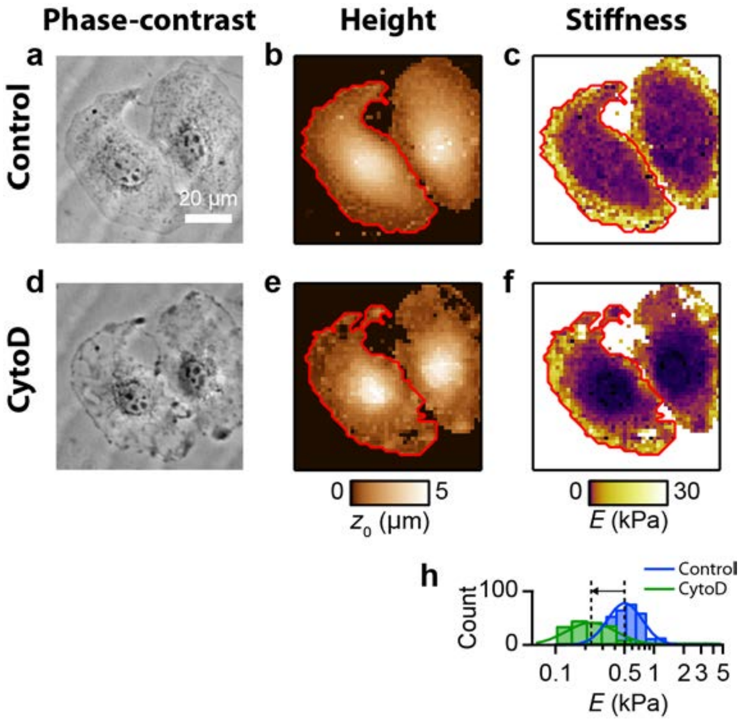

Figure Supplementary 3: AFM height and stiffness map of the same Ishikawa cell before and after 15 min treatment with cytochalasin D.

Optical phase-contrast image, AFM contact height image and AFM stiffness image of Ishikawa cell before (a-c) and 15 min after addition of cytochalasin D (d-f). The histograms of stiffness *E* (h) of the red outlined Ishikawa cell in (b,c) and in (e,f) shows a drastic shift of the stiffness distribution after cytochalasin D treatment, corresponding to a decrease in mean stiffness by 55%. The solid traces in the histogram show the log-normal distributions.

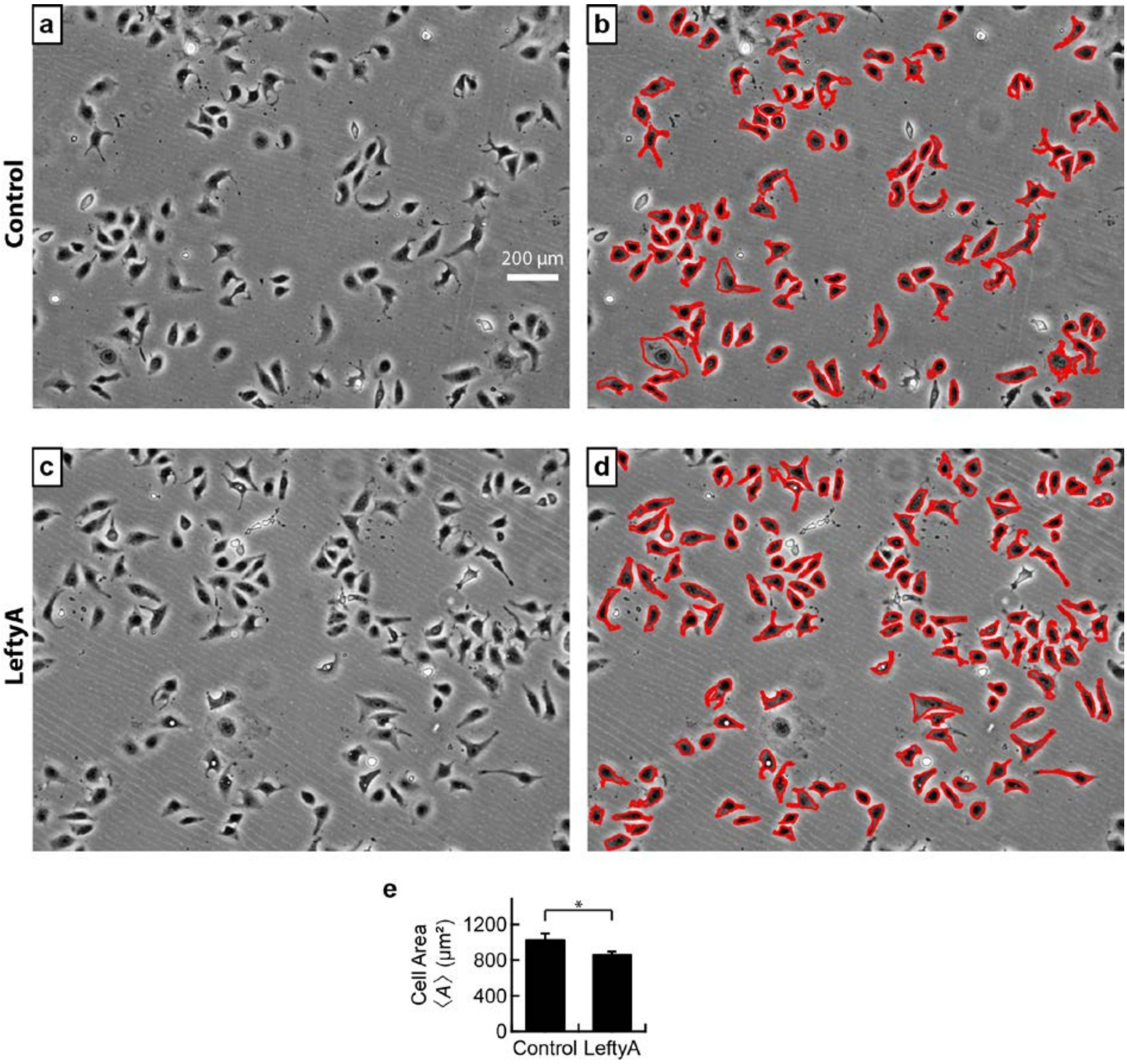

Figure Supplementary 4: Effect of LeftyA on cell morphology using optical phase-contrast microscopy.

Optical phase-contrast images using a 4x objective of Ishikawa cells before (a,b) and after (c,d) treatment with LeftyA. The red traces (b,d) show the outer contour of each cell, calculated by an automatic intensity threshold algorithm. Ishikawa cells treated by LeftyA exhibit a significantly lower mean cell area ($\langle A \rangle_{Contr}$ = 1038 µm², $\langle A \rangle_{LeftyA}$ = 872 µm²) (e). Error bars represent SEM of arithmetic mean. Data were tested for significance using Student's t-test (*, $P < 0.05$; **, $P < 0.01$; ***, $P < 0.001$).

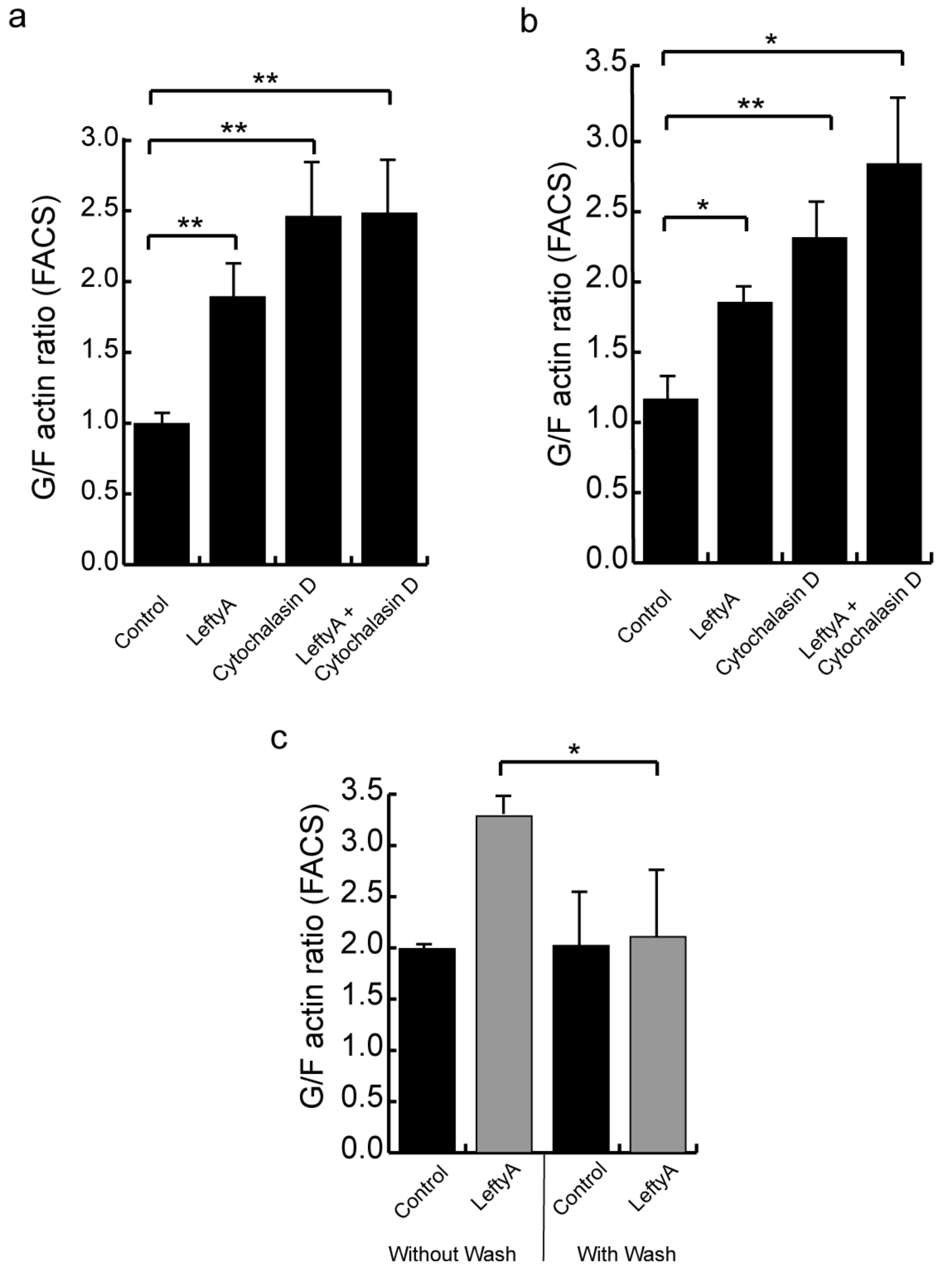

Figure Supplementary 5: Effect of LeftyA and CytochalasinD on actin polymerization in Ishikawa and HEK293 T-cells.

(a) Arithmetic means ± SEM (n = 4; arbitrary units) of soluble G-actin over filamentous F-actin ratio in Ishikawa cells remained untreated or treated with LeftyA (25 ng/ml) or cytochalasin D (10 µM) or in combination after for a 2 hours. (b) Arithmetic means ± SEM (n = 4; arbitrary units) of soluble G-actin over filamentous F-actin ratio in HEK293T cells remained untreated or treated with LeftyA (25 ng/ml) or cytochalasinD (10 µM) or in combination after for a 2 hours. (c) Arithmetic means ± SEM (n = 4; arbitrary units) of soluble G-actin over filamentous F-actin ratio in Ishikawa cells treated without and with LeftyA (25 ng/ml) after a 2 hour treatment (Left side) or treated without and with LeftyA (25 ng/ml) and then subsequently washed and placed in control medium for another 2 hours. *, $P < 0.05$; **, $P < 0.01$; ***, $P < 0.001$; Data were tested for significance using Student's t-test.

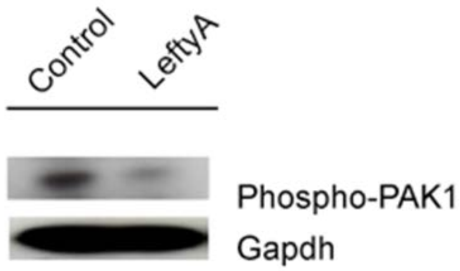

Figure Supplementary 6: LeftyA treatment decreases phospho PAK1 activity in Ishikawa cells.
Representative original Western blots showing phospho PAK1 protein abundance in human endometrial cancer Ishikawa cells after 2 hours culture in the absence or presence of LeftyA (25 ng/ml).

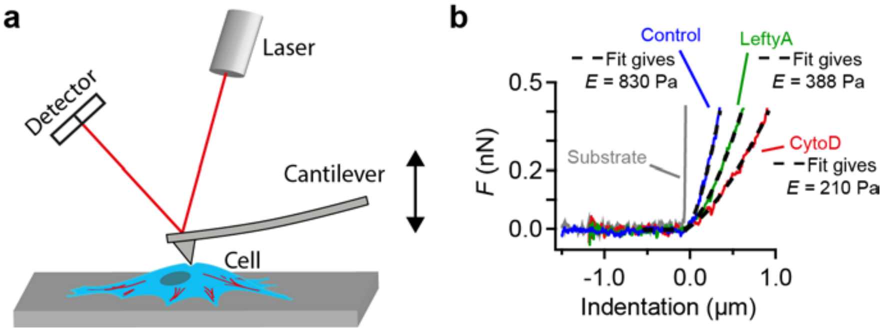

Figure Supplementary 7: AFM force measurement.
(a) Schematic of nanoscale atomic force microscopy (AFM) mechanical testing that was used to measure the stiffness of Ishikawa cells. (b) Representative force-indentation curves on a stiff substrate (grey), an untreated (blue), LeftyA (green) and cytochalasin D (red) treated Ishikawa cell. The force F is measured while approaching the cantilever toward the sample surface. After the cantilever tip contacts the sample surface the force increases rapidly on the stiff substrate, while it increases at a lower rate on a cell. A smaller increase corresponds to a smaller local stiffness. The local stiffness in terms of elastic modulus E was obtained by fitting the force-indentation curve with the spherical Hertz model.

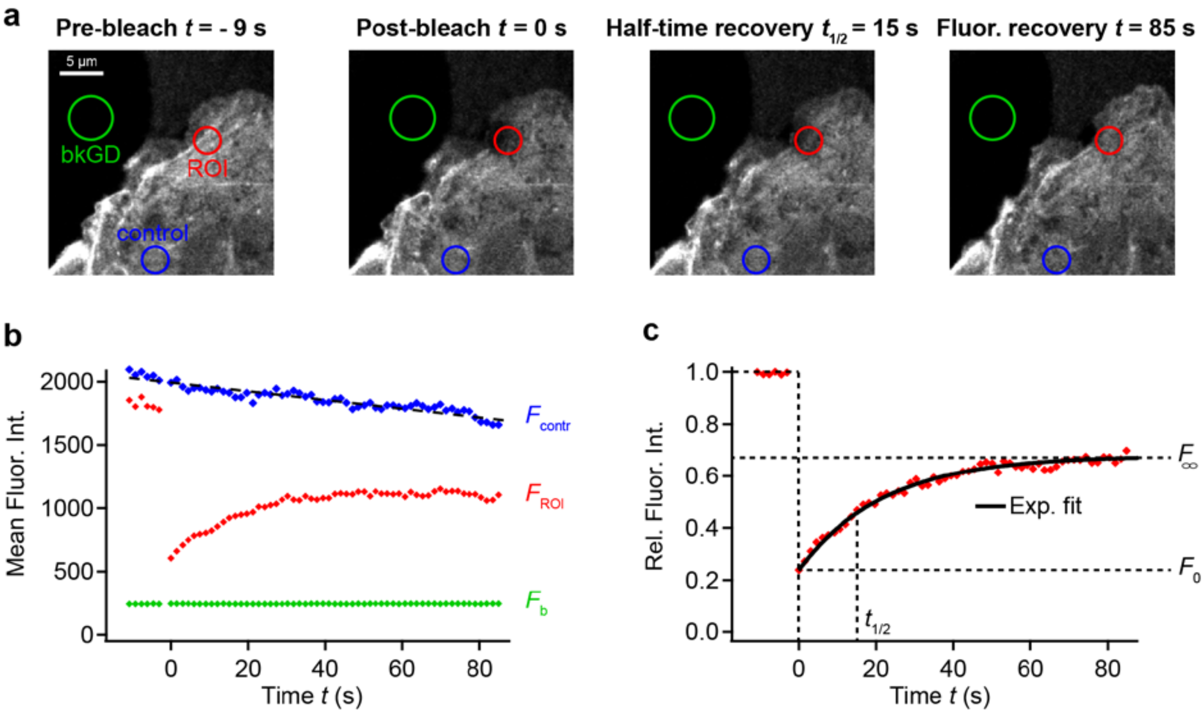

Figure Supplementary 8: FRAP assay.

(a) Confocal fluorescence images of an Ishikawa cell expressing GFP-actin during a FRAP experiment before bleaching (pre-bleach), immediately after bleaching the ROI (post-bleach), at the time $t_{1/2}$ when the half of the fluorescence intensity in the ROI had recovered and at the time of fluorescence recovery. (b) Mean fluorescence intensity of the control region, of the bleached ROI and of the background (bkGD). The dashed line indicates the overall photofading during image acquisition. (c) The background corrected, photofading corrected and normalized FRAP curve (using Eq. 2) was fitted by a one-phase exponential equation (Eq. 3) to obtain the half time of recovery $t_{1/2}$. F_0 and F_∞ are the normalized fluorescence intensities immediately after bleaching and after recovery, respectively.
